# Impact of low-dose ozone nanobubble treatments on antimicrobial resistance genes in pond water

**DOI:** 10.3389/fmicb.2024.1393266

**Published:** 2024-05-13

**Authors:** Qianjun Huang, Patrick Butaye, Pok Him Ng, Ju Zhang, Wenlong Cai, Sophie St-Hilaire

**Affiliations:** ^1^Department of Infectious Diseases and Public Health, Jockey Club College of Veterinary, City University of Hong Kong, Hong Kong, Hong Kong SAR, China; ^2^Department of Pathobiology, Pharmacology and Zoological Medicine, Faculty of Veterinary Medicine, Ghent University, Merelbeke, Belgium

**Keywords:** ozone treatments, disinfection, metagenomic shotgun sequencing, antimicrobial resistance genes, efflux mediated ARGs

## Abstract

Antimicrobial resistance (AMR) poses a significant global health threat as the silent pandemic. Because of the use of antimicrobials in aquaculture systems, fish farms may be potential reservoirs for the dissemination of antimicrobial resistance genes (ARGs). Treatments with disinfectants have been promoted to reduce the use of antibiotics; however, the effect of these types of treatments on AMR or ARGs is not well known. This study aimed to evaluate the effects of low dose ozone treatments (0.15 mg/L) on ARG dynamics in pond water using metagenomic shotgun sequencing analysis. The results suggested that ozone disinfection can increase the relative abundance of acquired ARGs and intrinsic efflux mediated ARGs found in the resistance nodulation cell division (RND) family. Notably, a co-occurrence of efflux and non-efflux ARGs within the same bacterial genera was also observed, with most of these genera dominating the bacterial population following ozone treatments. These findings suggest that ozone treatments may selectively favor the survival of bacterial genera harboring efflux ARGs, which may also have non-efflux ARGs. This study underscores the importance of considering the potential impacts of disinfection practices on AMR gene dissemination particularly in aquaculture settings where disinfectants are frequently used at low levels. Future endeavors should prioritize the evaluation of these strategies, as they may be associated with an increased risk of AMR in aquatic environments.

## 1 Introduction

The persistence and spread of antimicrobial resistance (AMR) in the environment, is considered a global silent pandemic and one of the leading health issues facing the world (Mendelson et al., 2022). It is estimated that AMR leads to approximately 700,000 deaths annually and the number is projected to rise to 10 million deaths per year in 2050 (Joshua et al., 2022). Additionally, by 2050, AMR could cost $100 trillion in lost economic output (Burki, 2018). Given the use of antibiotics in aquaculture, fish farms have been suggested as potential sources and sinks for antimicrobial resistance (Watts et al., 2017). Addressing AMR associated with aquaculture practices requires a comprehensive approach, including setting standards for antibiotic use, promoting responsible and prudent use of antibiotics, and adopting alternative disease management strategies and biosecurity to prevent disease outbreaks and reduce reliance on these drugs (Bhat and Altinok, 2023).

One of the strategies commonly used in fish farming to decrease the use of antibiotics is treatment with low dose disinfectants (Rico and Van den Brink, 2014). These disinfectants can help decrease bacterial pathogens on the fish or in the water column (Hushangi and Hosseini Shekarabi, 2018), thereby lowering the risk of systemic infections and minimizing antibiotic dependence. Topical disinfectants can be administered in aquaculture systems continuously, as low dose indefinite baths, or at higher dose as short-term dips (Mota et al., 2022). Regardless of the strategy used, the process rarely eliminates all the bacteria in the system. The remaining bacteria have the potential to regrow and drive the microbial community (Pei et al., 2019; Huang et al., 2023).

It has been shown that bacteria that remerge after disinfection may be more resistant not only to the disinfectant used, but also to antibiotics (Schweizer, 2001; McBain et al., 2002; Sharma et al., 2016). The mechanisms for this selection have still to be elucidated, but specific gene mutations, changes in cell envelope permeability, increased expression of efflux pump, and increased ability to form biofilms have been hypothesized (McDonnell and Russell, 1999; Poole, 2002; Rozman et al., 2021). These findings highlight the importance of considering the potential impacts of disinfection on ARGs particularly in aquaculture where disinfection is used frequently. The objective of this study was to evaluate the effect of low dose ozone on the relative abundance of ARGs in pond water using shotgun metagenomic sequencing analysis.

## 2 Materials and methods

### 2.1 Experimental design

The experiment was conducted in August 2022 at the Au Tau Fisheries Office, Agriculture, Fisheries and Conservation Department in Yuen Long, Hong Kong SAR, PR China. The pond water we used had not been treated with any antibiotics and the last H_2_O_2_ (7 mg/L) treatment for algae control was done over a year prior to our study. The original aim of the study was to assess the effect of different ozone delivery systems on the microbial communities. The detailed methods and results of the initial study are published elsewhere (Huang et al., 2023). We used the sequence data from this prior study to subsequently assess the effect of ozone on the relative abundance of ARGs. In brief, pond water was pumped into twelve 75L tanks. There were 4 groups in total, two ozone treatment groups, ozone macrobubbles (O3MB) and ozone nanobubbles (O3NB), and two control groups, air macrobubbles (AirMB) and air nanobubbles (AirNB). All groups were triplicated.

To generate ozone nanobubbles, a nanobubbler (aQua+075MO, AquaPro Solutions Pte Ltd., Singapore) was connected to an ozone generator (DNO-15G, Dino Purification Co., Ltd, China) with an incoming oxygen stream flow rate of approximately 0.3 LPM. Water from the tanks was circulated into the nanobubbler and subsequently reintroduced back into the tanks. A dissolved ozone meter (DOZ-30, Dino Purification Co., Ltd, China) was used to monitor the ozone concentration meanwhile, and the system was stopped when the desired ozone concentration of 0.15 mg/L was attained in the three O3NB tanks. the same ozone concentration of 0.15 mg/L was achieved in O3MB group by attaching the same ozone generator to an air stone to deliver gaseous ozone macrobubbles to the tanks. For the AirMB and AirNB group, air pumps (Dazs model AP-528, Hong Kong) with a flow rate of 1.2 L/min were attached to air stones and the nanobubbler, respectively to provide microbubble and nanobubble aeration, and these were run for the same amount of time as the pumps used in the O3MB and O3NB treatment group, respectively.

Water samples (400 mL) were collected before and 24 h after the different treatments, and ozone was not detected at either of these time points (0 mg/L). Water was filtered, DNA was extracted using QIAamp Fast DNA Stool Mini Kit (Qiagen™), and then submitted to Novogene for 150 bp pair-end metagenomic shotgun sequencing. A sequencing depth of 6 Gb (Giga base pairs) was applied for each sample on Illumina HiSeq platform.

### 2.2 Metagenomic data processing

After the sequencing, raw reads were cleaned using Readfq (V8).^1^ The clean data were assembled using MEGAHIT software (v1.0.4-beta) (Li et al., 2015). Fragments shorter than 500 bp in the contigs were removed from the assembly. MetaGeneMark (v2.10) (Gemayel et al., 2022) was then used to predict genes on contigs, and the information with a length less than 100 nt in the prediction results was filtered out. We used the CD-HIT software (v4.5.8)^2^ with the default setting to create a unique initial gene catalog. Clean data of each sample was aligned to this initial gene catalog to determine the number of reads that matched the genes in our catalog using Bowtie2 (Bowtie2.2.4). Genes with reads ≤2 in each sample were filtered out. The filtered data was used for identification of ARGs and subsequent analysis. Based on the number of reads aligned and the length of the genes, the relative abundance of each gene in each sample was calculated by the following formula (Cotillard et al., 2013; Le Chatelier et al., 2013; Zeller et al., 2014), in which r was the number of gene reads, and L is the length of gene.

$$G_{k}=\frac{r_{k}}{L_{k}}\cdot \frac{1}{\sum_{i=1}^{n}\frac{r_{i}}{L_{i}}}$$

Notes: “k” represents a certain gene and “i” was a value from 1 to n, used to cycle through all genes.

### 2.3 Bacterial taxonomy and ARGs analysis

The presence of acquired antimicrobial resistance genes was determined using the ResFinder tool (Florensa et al., 2012). Because we did not know the bacterial origin of most of our genes it was sometimes difficult to classify genes as acquired or intrinsic in this database as in cases such as (*bla*_*PAO*_, *bla*_*SGM*_ and *oleB*) the nature of the gene could differ depending on the bacterial origin. In these cases, we classified the gene as acquired because it has been described in the literature as acquired in at least one bacterial species. To find the intrinsic ARGs belonging to the following 4 families, ATP binding cassette (ABC), resistance nodulation cell division (RND), small multidrug resistance (SMR), and major facilitator super (MFS) family, we used the CARD-RGI tool (McArthur et al., 2013). The threshold values for inclusion in our study were, *e*-value < 1e−9, identity >70%, and alignment length >100 bp. The Comprehensive Antibiotic Resistance Database (CARD) was also used to determine the mechanism of action and the antibiotic class that the genes conferred to.

DIAMOND (Version 0.9.9.110) was utilized to search genes with the *e*-value threshold of 1e−5 against sequences of bacteria, fungi, archaea, and viruses obtained from the NR database of the National Center for Biotechnology Information (NCBI). We used the LCA algorithm to ensure accurate species annotation. Finally, we identified whether the ten most abundant bacterial genera, which dominated post treatment had either intrinsic or acquired AMR genes associated with efflux pump proteins.

### 2.4 Statistical analysis

All data, including specific acquired ARGs, as well as their associated antibiotic classes, and resistance mechanisms, were graphically represented using box plots and stacked bar plots. The data were expressed as mean ± the standard deviation. To assess significant changes in relative abundance between pre- and 24-h post-treatments within treatment groups, we performed paired *t*-tests after log-transforming the data when the model residuals did not meet the assumptions of normality and homogeneity of variance. A *p*-value threshold of less than 0.05 was used to determine statistical significance. We also evaluated the difference in the relative abundance of intrinsic resistance genes for efflux protein families, RND, ABC, SMR and MFS, between pre and 24 h-post treatment in the four treatment groups using the same statistical methods. All statistical analysis were done in R Statistical Software (v4.1.2) (R Core Team, 2021).

A dendrogram was used to visualize the hierarchical cluster analysis, which was performed using Ward’s clustering method with squared Euclidean distance to determine if the acquired ARG profiles between treatments were different (Ward, 1963).

Additionally, we investigated whether acquired or intrinsic ARGs co-existed within the same genera of bacteria for overall and post-ozone treated samples by using a Sankey chart, which linked the relationships between these ARGs and the bacterial genera, while highlighting the interplay of resistance mechanisms. This was done using the “networkD3” package in the R Statistical Software (v4.1.2) (R Core Team, 2021).

## 3 Results

### 3.1 Descriptive metagenomic data

We obtained an average of 43,673,320 ± 260,619 paired-end reads per water sample. After quality control, we retained 43,584,916 ± 261,566 paired-end reads per sample, generating a total of 21,792,458 ± 130,783 merged reads. The details of the quality control steps are outlined in Huang et al. (2023). The processed reads were on average assembled into 251,556 contigs per sample with an average length of 1,208 bp (Supplementary Table 1). These reads were used to identify the ARGs in the pre and post air or ozone treated water samples.

### 3.2 Antimicrobial resistant genes analysis

Based on shotgun metagenomic analysis, there were 26 different acquired ARGs identified within the predicted open reading frames (ORFs), which were associated with resistance against ten different classes of antibiotics and encompassed five resistance mechanisms (Supplementary Table 2).

#### 3.2.1 Acquired antibiotic resistance genes

**FIGURE 1:**
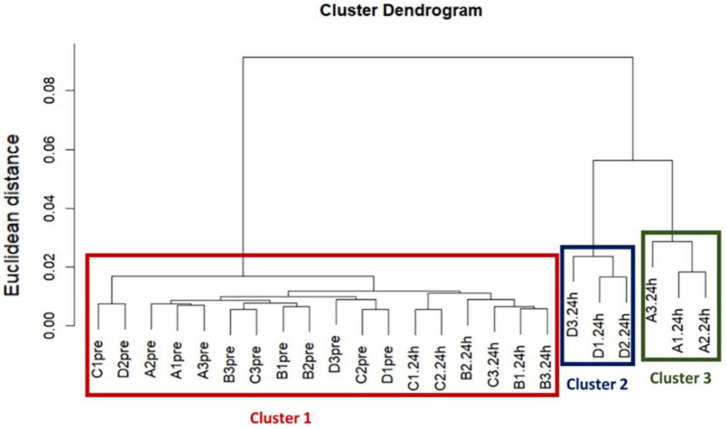
The results of a Hierarchical cluster analysis on acquired resistance genes revealed three distinct clusters corresponding to the two ozone treatments and the control air treatment groups. Treatments are labeled as, A: ozone macrobubble group (O3MB), B: air macrobubble group (AirMB), C: air nanobubble group (AirNB) and D: ozone nanobubble group (O3NB). The number following is the replicate, followed by the sampling time, pre: pretreatment sample, 24 h: sample after 24 h of the treatment. Our analysis showed all pre-treatment samples and 24 h-post control air treatment samples clustered together in the Cluster 1, and the three samples from the 24 h-post ozone nanobubble treated group were categorized into cluster 2. Cluster 3 was comprised by three samples in 24 h-post ozone macrobubble treated group.

**FIGURE 2:**
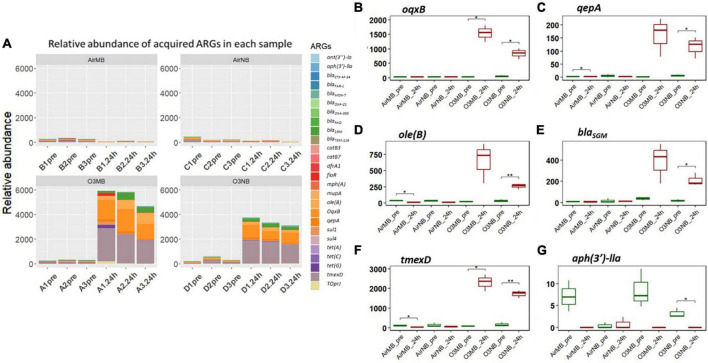
**(A)** Relative abundance of acquired ARGs of each sample across four treatments; **(B–G)** Relative abundance of specific ARGs before (pre) and 24 h after (24 h) air or ozone treatments. Significant difference in the relative abundance of ARGs between pre and post treatment samples were determined using a Paired *T*-test and is indicated with *. Only one ARG showed a significant decrease. *Y*-axis represents relative abundance of each gene. *X*-axis represents sample name, in which A is ozone macrobubble group (O3MB), B is air macrobubble group (AirMB), C is air nanobubble group (AirNB) and D is ozone nanobubble group (O3NB). The number following is the replicate, followed by the sampling time, pre, pretreatment sample; 24 h, sample after 24 h of the treatment.

Our hierarchical clustering analysis on the acquired ARGs revealed three distinct clusters corresponding to the two ozone treatments and the air treatment groups (controls) (Figure 1). In summary, all pre-treatment samples and 24 h-post air treated samples clustered together (Cluster 1), and the 24h-post treated samples in the two ozone groups clustered separately based on the type of bubbles used to deliver the gas (Cluster2 & 3). The clustering was mainly driven by the increase in relative abundance of five genes *oqxB*, *qepA, oleB*, *bla*_*SGM*_ and *tmexD* (Figures 2A–F).

In contrast, the *aph(3′)-IIa* gene showed a significant decrease following ozone nanobubble (O3NB) treatment compared to the air treatment groups (Figure 2G). Although there was a trend suggesting the ozone macrobubble (O3MB) treated water also had a reduction in this particular gene compared to the O3NB group, this difference was not statistically significant (Figure 2G).

#### 3.2.2 Acquired genes grouped by antibiotic classes

**FIGURE 3:**
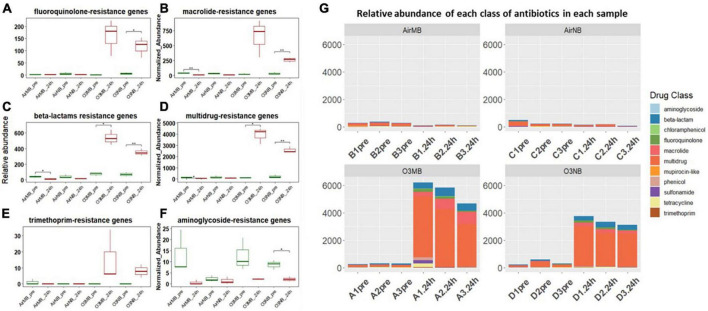
**(A–F)** Relative abundance of ARGs grouped by antibiotic classes before (pre) and 24 h after (24 h) air or ozone treatments. Significant difference in the relative abundance of genes in different antibiotic classes between pre and post treatment samples were determined using a Paired *T*-test and is indicated with *. Only one class showed a in significant decrease; **(G)** Normalized abundance of antibiotics classes of each sample across four treatments, which showed ARGs associated with multidrug resistance increased and dominated in the community after the ozone treatments. *Y*-axis represents relative abundance of each antibiotic class. *X*-axis represents sample name, in which A is ozone macrobubble group (O3MB), B is air macrobubble group (AirMB), C is air nanobubble group (AirNB) and D is ozone nanobubble group (O3NB). The number following is the replicate, followed by the sampling time, pre, pretreatment sample; 24 h, sample after 24 h of the treatment.

Our analysis of the acquired ARGs categorized by the class of antibiotics they affect, suggested a notably increase in the relative abundance of genes associated with resistance to the following classes of drugs: fluoroquinolones, macrolides, and beta-lactam antibiotics in the 24 h-post ozone treated groups compared to the controls (Figures 3A–C). In addition, the genes associated with resistance to multidrugs accounted for over 70% of ARGs after treatment with ozone (Figures 3D, G). While there was an observable increase in trimethoprim-resistance genes in the ozone treated groups, the difference between pre- and 24 h-post treatment was not statistically significant (Figure 3E). Conversely, there was a decrease in the genes encoding aminoglycoside-resistance after ozone treatments; however, this decrease was only significant in the O3NB group (Figure 3F).

#### 3.2.3 Acquired genes grouped by mechanisms

**FIGURE 4:**
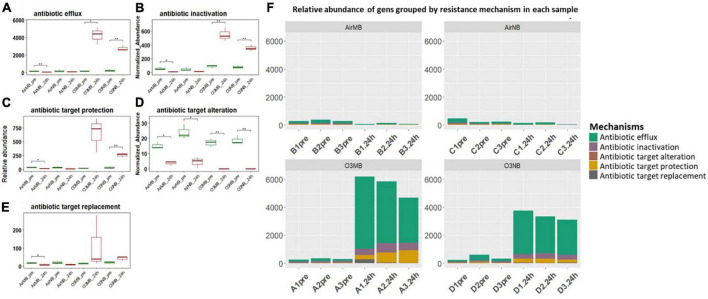
**(A–E)** Relative abundance of ARGs grouped by resistance mechanisms before (pre) and 24 h after (24 h) air or ozone treatments. Significant difference in the relative abundance of genes in different resistance mechanisms between pre and post treatment samples were determined using a Paired *T*-test and is indicated with *. Antibiotic target alteration showed a significant decrease after treatments in all groups; **(F)** Relative abundance of genes grouped by resistance mechanism in each sample across four treatments, which showed ARGs associated with antibiotic efflux mechanism increased and dominated in the community after the ozone treatments. *Y*-axis represents relative abundance of genes grouped by mechanism. *X*-axis represents sample name, in which A is ozone macrobubble group (O3MB), B is air macrobubble group (AirMB), C is air nanobubble group (AirNB) and D is ozone nanobubble group (O3NB). The number following is the replicate, followed by the sampling time, pre, pretreatment sample; 24 h, sample after 24 h of the treatment.

The acquired ARGs identified in our study were also categorized into five primary resistance mechanisms, which included antibiotic inactivation, antibiotic efflux systems, antibiotic target alternation, antibiotic target protection, and antibiotic target replacement (Figures 4A–E and Supplementary Table 2). When we grouped the genes by their mechanisms of action, we observed significant increases in the relative abundance of ARGs encoding for the antibiotic efflux systems, antibiotic inactivation, and antibiotic target protection following the ozone treatments (Figures 4A–C, F). In contrast, the genes associated with antibiotic target alteration, initially present at low levels, exhibited a significant decrease in relative abundance after all treatments (Figure 4D).

#### 3.2.4 Intrinsic efflux genes

**FIGURE 5:**
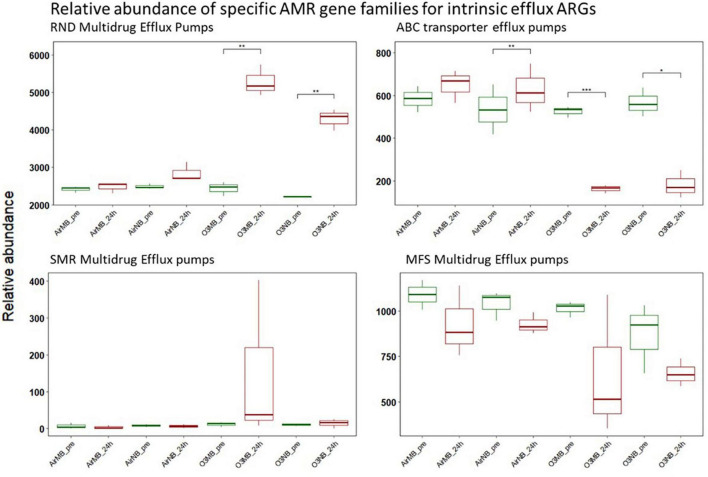
Relative abundance of specific AMR gene families for intrinsic efflux ARGs before (pre) and 24 h after (24 h) air or ozone treatments. Significant difference in the relative abundance of genes in different families between pre and post treatment samples were determined using a Paired *T*-test and is indicated with *. Only genes in the RND family showed a significant increase in relative abundance after ozone treatments, while ABC family decreased significantly after ozone treatments. *Y*-axis represents relative abundance of genes grouped by mechanism. *X*-axis represents sample name, in which AirMB is air macrobubble group, AirNB is air nanobubble group, O3MB is ozone macrobubble group and O3NB is ozone nanobubble group. The number following is the replicate, followed by the sampling time, pre, pretreatment sample; 24 h, sample after 24 h of the treatment.

**FIGURE 6:**
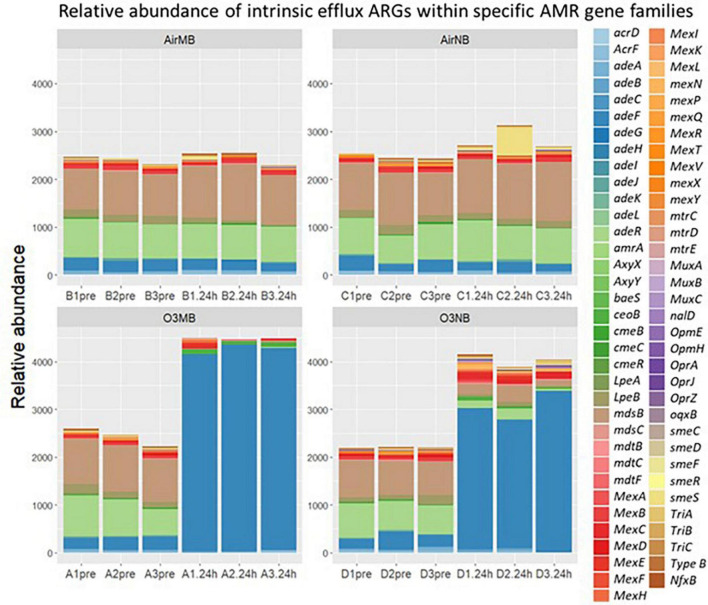
Relative abundance of intrinsic efflux ARGs within specific AMR gene families including ATP binding cassette (ABC), resistance nodulation cell division (RND), small multidrug resistance (SMR), and major facilitator super (MFS) family detected by CARD-RGI identifier before (pre) and 24 h after (24 h) air or ozone treatments delivered by macrobubbles or nanobubbles, which showed adeF gene became the most abundant intrinsic efflux ARGs in the water samples 24 h after the ozone treatments. *Y*-axis represents relative abundance of genes. *X*-axis represents sample name, in which A is ozone macrobubble group (O3MB), B is air macrobubble group (AirMB), C is air nanobubble group (AirNB) and D is ozone nanobubble group (O3NB). The number following is the replicate, followed by the sampling time, pre, pretreatment sample; 24 h, sample after 24 h of the treatment.

The intrinsic efflux ARGs, which were identified using the CARD-RGI were initially more abundant in all samples compared to the acquired ARGs. Similar to the change in relative abundance of acquired efflux ARGs, the intrinsic efflux ARGs from the RND family were significantly higher in ozone treated samples compared to air treated samples 24 h after the treatment (Figure 5), with the *adeF* gene becoming the most abundant intrinsic efflux ARGs in RND family in the water samples post ozone treatments (Figure 6). However, the intrinsic efflux ARGs in the ABC family significantly decline after ozone treatments, which was driven mainly by *mel, tlrC*, and *otrC* (Figure 5).

#### 3.2.5 ARGs linked to specific bacterial genera

**FIGURE 7:**
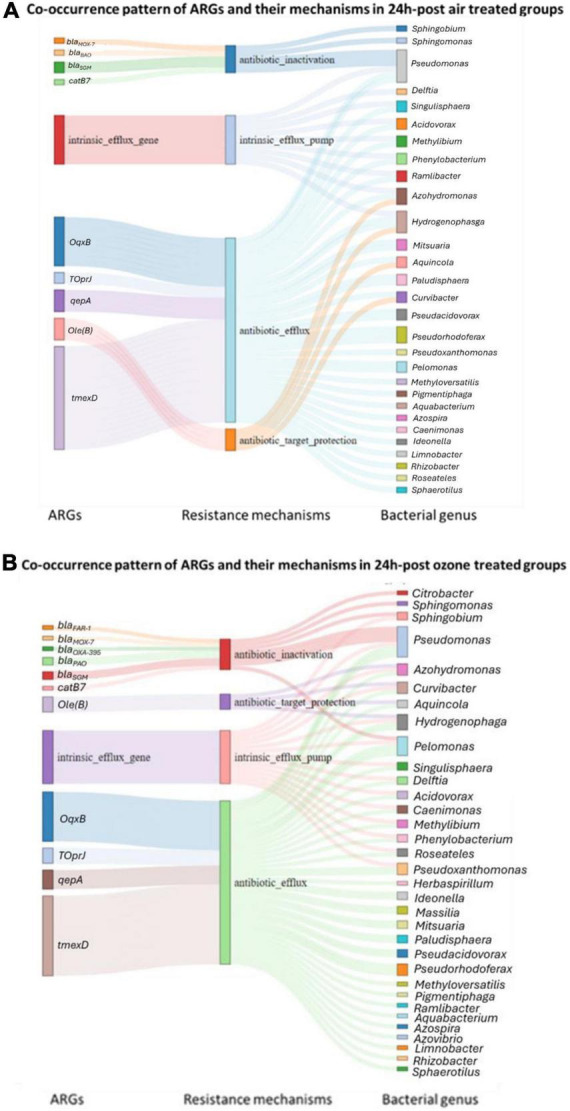
Sankey diagram used to investigate whether acquired ARGs or intrinsic efflux ARGs co-existed within the same known genera of bacteria 24 h after air treatment ed samples **(A)** and ozone treatment **(B)**, while highlighting the interplay of resistance mechanisms. Regardless of the treatment efflux pump ARGs were linked to almost all bacterial genera with the exception of 2 or 3, suggesting ozone treatment did not alter the co-existence pattern of ARGs.

**FIGURE 8:**
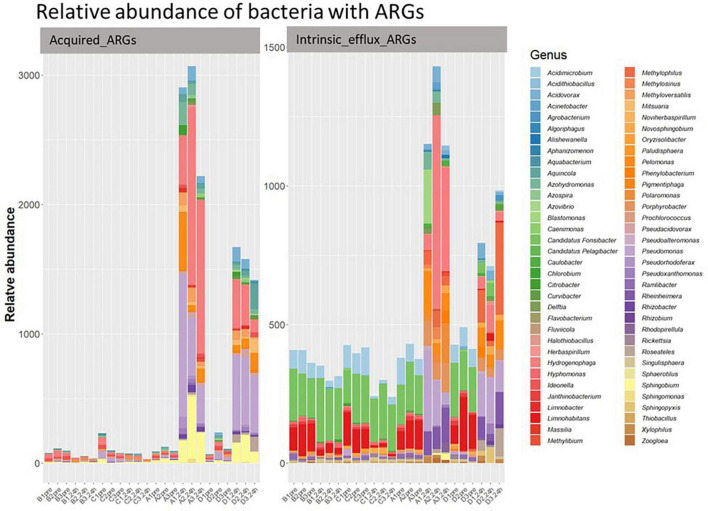
The relative abundance of classified bacterial genera with acquired efflux ARGs (left) and intrinsic efflux ARGs (right) in all samples. *Y*-axis represents relative abundance of bacteria with ARGs. *X*-axis represents sample name, in which A is ozone macrobubble group; B is air macrobubble group; C is air nanobubble group and D is ozone nanobubble group. The number following is the replicate, followed by the sampling time, pre, pretreatment sample; 24 h, sample after 24 h of the treatment.

Of the acquired ARGs identified in our study, only 9.63% were classified to 33 of the 2,552 bacterial genera present in our samples (Figure 7). The other acquired ARGs could not be linked to specific bacteria in our sample, though this could be because there was insufficient information in our database to classify their origin. Further, 44 genera of bacteria in our samples had genes belonging to the four intrinsic efflux pump families. The relative abundance of the bacterial genera associated with (acquired or intrinsic) ARGs increased post ozone treatments, while bacteria genera associated with ARGs did not increase in relative abundance after the air treatment samples (Figure 8).

Most of the 33 genera in our study had at least one gene associated with acquired efflux pumps (Figure 7). For example, the *tmexD*, *oqxB*, *qepA*, and *toprJ*, four efflux pump genes, were present in 21, 13, 5, and 4 bacterial genera (Figure 7), respectively, and all of these genes increased after ozone treatment (Figure 2). Interestingly, of the 10 most abundant bacterial genera (Supplementary Figure 1) in our post-ozone treated samples, all had at least eight efflux ARGs (Supplementary Table 3). In contrast, the number of genera in the 24 h-post air treated groups that had efflux pump ARGs was at most 6 (Supplementary Table 3).

It is worth noting that after the ozone treatments, a number of specific non-efflux ARGs of importance (i.e. beta-lactam-resistance genes (*bla*_*OXA–395*_, *bla*_*MOX–7*_ and *bla*_*PAO*_), chloramphenicol resistance genes (*catB7*) remained in the bacterial population (Figure 7B). However, these genes were always in genera that also had efflux pump genes with the few exceptions of *Citrobacter* and *Sphingomonas.*

## 4 Discussion

Our shotgun metagenomic analysis of pond water treated with low dose ozone (0.15 mg/L) indicated that a one-time treatment, irrespective of how this gas was delivered (nanobubbles or macrobubbles), played a role in influencing the composition and relative abundance of antimicrobial resistant genes (ARGs) 24 h after treatments. Our findings suggested an increase in intrinsic efflux and acquired ARGs. Five out of 33 acquired ARGs detected in our samples increased significantly in relative abundance 24 h after a low dose ozone treatment. These 5 genes were associated with antibiotic efflux, antibiotic activation, and antibiotic target protection mechanisms; however, the efflux pump genes appeared to be more prominent in the ten most abundant bacterial genera post treatment. Other researchers have also reported similar ARG changes in bacterial communities exposed to different disinfectants, such as chlorine, hydrogen peroxide, quaternary ammonium, peracetic acid, chlorhexidine diacetate and trisodium phosphate (Su and D’Souza, 2012; Capita et al., 2013; Biswal et al., 2014; Molina-González et al., 2014; Gadea et al., 2016). Interestingly, we found an increase in relative abundance for a few specific non-efflux ARGs after ozone treatments, but closer inspection revealed that they usually co-existed in bacterial genera associated with intrinsic or acquired efflux ARGs.

The increased relative abundance of both intrinsic efflux and acquired ARGs associated with an efflux pump mechanism after treatment with ozone was somewhat expected. Many of these genes play a crucial role in the survival of bacteria, particularly Gram-negative bacteria, by expelling toxic substances, including antibiotics and other compounds such as those generated during oxidative stress (Nelson, 2002; Huwaitat et al., 2016; Masi et al., 2017). In fact, many researchers have found both acquired efflux pump genes such as *qepA*, *tet(G)* and *oqxB*, and the intrinsic efflux genes in the RND family to be up-regulated in response to different stressors, suggesting they play an important role in bacterial survival (Shi et al., 2013; Tandukar et al., 2013; Xu et al., 2017; Gu et al., 2020; Hu et al., 2023; McCarlie et al., 2023). Therefore, it is not surprising that we detected an increase in the relative abundance of genes associated with the efflux pump mechanism after exposure to low dose ozone, which is associated with oxidative stress (Pell et al., 1997).

It is important to better understand the selective pressure that ozone exerts on bacterial communities at different doses used in aquaculture particularly low dose treatments that may not kill all bacteria (Iakovides et al., 2019, 2021; Huang et al., 2023). Our study suggests at lower ozone concentrations, which are used to treat fish, bacteria are not completely eliminated (Huang et al., 2023), and the surviving bacteria may be more resistant to ozone. This shift in population could have additional consequences on other types of treatments, particularly antibiotic therapies as the genes suspected of conferring ozone resistance may also bestow resistance to antibiotics.

Besides an increase in ARGs associated with efflux pumps, we also observed a significant increase in the relative abundance of a few non-efflux genes either involved in antibiotic target protection or antibiotic inactivation after our ozone treatments. Previous studies also found an increase in antibiotic specific genes in wastewater after treatment of with 0.9 ± 0.1 g ozone per 1 g DOC. In one study, there was an increase in the abundance of *vanA* and *bla*_*VIM*_ genes, encoding vancomycin and carbapenem resistance (Alexander et al., 2016). Another study found after an ozone treatment, the surviving *R. ornithinolytica* bacterial population had an increase in the *bla*_*CTX–M–62*_ gene (Azuma et al., 2022). It is possible genes that conferred ozone resistance in our study (i.e., efflux pump genes) co-occurred with AMR specific (i.e., non-efflux pump) genes, which could therefore lead to their co-selection under ozone exposure. Co-selection of genes under different environment stressors has been reported by others (Ding et al., 2019). In our study, there was evidence for this hypothesis as we found several non-efflux pump ARG often co-occurring within the same genera of bacteria as acquired efflux ARGs (i.e., *OqxB*) or the intrinsic RND efflux genes (i.e., *adeF*). In fact, all but two of the ten most abundant bacteria that survived and colonized our samples after an ozone treatment had either intrinsic RND efflux genes or acquired efflux ARGs. Interestingly, these two genera *Sphingomonas and Citrobacter*, are known to form biofilms. This protective mechanism protects bacteria against disinfectants (Donlan and Costerton, 2002; Gulati and Ghosh, 2017) and may explain their survival post ozone treatment.

Notably, a few ARGs such as *dfrA1*, *floR* and *ant(3″)-Ia*, which increased in relative abundance after 24 h after ozone treatments are known to be located on mobile genetic elements (MGEs) (Zhang et al., 2017; Sánchez-Osuna et al., 2020; Raza et al., 2022; Zhu et al., 2022). If the increased ARGs observed in our study post treatment were present within MGEs, the enrichment of those ARGs could lead to nonpathogenic microbial species acting as an ecological reservoir for pathogenic bacteria (Peterson and Kaur, 2018). Bacteria can acquire new ARGs through horizontal gene transfer from plasmids, transposons, or other MGEs originating from other bacteria or the environment (Jian et al., 2021).

This study had several limitations that should be acknowledged. Firstly, our results were based on only one pond. Different pond water sources could be included in future studies to increase the external validity of the findings. Secondly, we used shotgun metagenomic analysis which only provided us with short-read sequencing. This is particularly problematic for determining the origin of genes within the mixed bacterial community. Despite attempts to address this limitation by employing read assembly techniques (Li et al., 2022), we could not determine the source of many ARGs (i.e., bacterial genomic or MGEs). Another limitation is that the classifiers we used to determine whether genes were intrinsic or acquired were not consistent across all bacterial genera. There were some genes (i.e., *bla*_*PAO*_, *bla*_*SGM*_, *oleB*) that are likely intrinsic for some bacteria and considered acquired for others, therefore, it was difficult for us to categorize these as we did not know the bacterial origin of all the genes. Despite this limitation, of the genes we classified as acquired (*n* = 33), there were only a few which could have been intrinsic depending on the origin (Li et al., 2022). Finally, it is important to note that our reporting of relative changes in certain genes between samples, was achieved by normalizing the reads to the total sequence reads, therefore we could only assess relative abundance. This measure does not necessarily translate to phenotypic antibiotic resistance or absolute abundance. To investigate the absolute abundance of ARGs, quantitative polymerase chain reaction (qPCR) analyses would be required, and this was beyond the scope of this study.

## 5 Conclusion

In this study, we provide valuable insights into the effects of low-dose ozone treatments on ARG dynamics in aquaculture pond water. The results indicate that low dose ozone disinfection, delivered through macrobubbles or nanobubbles, can lead to an increase in the relative abundance of ARGs. We saw the increase mainly with the efflux pumps, whether acquired or naturally present in the bacteria. We also have evidence to suggest co-selection of other resistance genes. This study emphasizes the importance of considering the potential impacts of disinfection on ARGs, particularly in aquaculture settings where disinfectants are frequently used at low levels.

## Data availability statement

The datasets presented in this study can be found in online repositories. The names of the repository/repositories and accession number(s) can be found below: https://www.ncbi.nlm.nih.gov/bioproject/1100576.

## Author contributions

QH: Writing – review and editing, Writing – original draft, Visualization, Methodology, Investigation, Formal analysis, Data curation, Conceptualization. PB: Writing – review and editing, Methodology, Formal analysis. PN: Writing – review and editing, Investigation. JZ: Writing – review and editing, Investigation. WC: Writing – review and editing, Investigation. SS-H: Writing – review and editing, Writing – original draft, Supervision, Project administration, Investigation, Funding acquisition.

## Footnotes

1
https://github.com/cjfields/readfq

2
http://www.bioinformatics.org/cd-hit/

## Supplementary material

Supplementary Figure 1 Relative abundance of the top 10 genera present in the microbial communities before (pre) and 24 h after (24 h) air or ozone treatments delivered by macrobubbles or nanobubbles. *Y*-axis represents accumulated proportion of the genera. *X*-axis represents sample name, in which A is ozone macrobubble group, B is air macrobubble group, C is air nanobubble group and D is ozone nanobubble group.
