# Supplementary figures and images for: Impact of low-dose ozone nanobubble treatments on antimicrobial resistance genes in pond water
Source: Front Microbiol. 2024 May 13;15:1393266. doi: 10.3389/fmicb.2024.1393266 (PMC11136503; doi:10.3389/fmicb.2024.1393266)

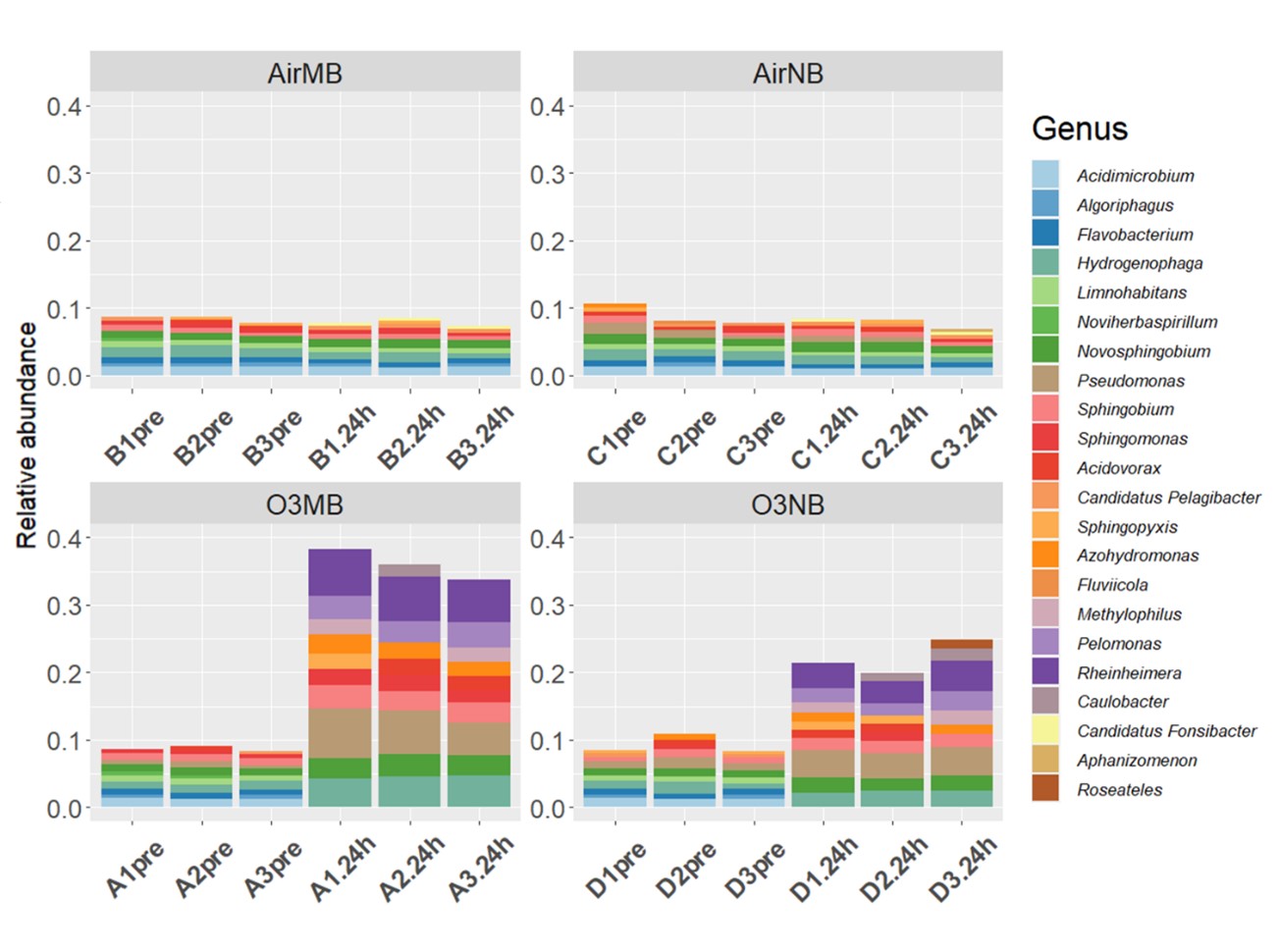

Supplement: Supplementary Figure 1 — Relative abundance of the top 10 genera present in the microbial communities before (pre) and 24 h after (24 h) air or ozone treatments delivered by macrobubbles or nanobubbles. Y-axis represents accumulated proportion of the genera. X-axis represents sample name, in which A is ozone macrobubble group, B is air macrobubble group, C is air nanobubble group and D is ozone nanobubble group. [file Image_1.JPEG]
